# Increased Postprandial Nonesterified Fatty Acid Appearance and Oxidation in Type 2 Diabetes Is Not Fully Established in Offspring of Diabetic Subjects

**DOI:** 10.1371/journal.pone.0010956

**Published:** 2010-06-04

**Authors:** François Normand-Lauzière, Frédérique Frisch, Sébastien M. Labbé, Patrick Bherer, René Gagnon, Stephen C. Cunnane, André C. Carpentier

**Affiliations:** 1 Division of Endocrinology, Department of Medicine, Université de Sherbrooke, Sherbrooke, Québec, Canada; 2 Division of Genetics, Department of Pediatrics, Université de Sherbrooke, Sherbrooke, Québec, Canada; 3 Research Center on Aging, Sherbrooke, Québec, Canada; Mayo Clinic College of Medicine, United States of America

## Abstract

**Background:**

It has been proposed that abnormal postprandial plasma nonesterified fatty acid (NEFA) metabolism may participate in the development of tissue lipotoxicity and type 2 diabetes (T2D). We previously found that non-diabetic offspring of two parents with T2D display increased plasma NEFA appearance and oxidation rates during intravenous administration of a fat emulsion. However, it is currently unknown whether plasma NEFA appearance and oxidation are abnormal during the postprandial state in these subjects at high-risk of developing T2D.

**Methodology:**

Palmitate appearance and oxidation rates and glycerol appearance rate were determined in eleven healthy offspring of two parents with T2D (positive family history, FH+), 13 healthy subjects without first-degree relatives with T2D (FH-) and 12 subjects with T2D at fasting, during normoglycemic hyperinsulinemic clamp and during continuous oral intake of a standard liquid meal to achieve steady postprandial NEFA and triacylglycerols (TG) without and with insulin infusion to maintain similar glycemia in all three groups.

**Principal Findings:**

Plasma palmitate appearance and oxidation were higher at fasting and during the clamp conditions in the T2D group (all *P*<0.05). In the postprandial state, palmitate appearance, oxidative and non oxidative rates were all elevated in T2D (all *P*<0.05) but not in FH+. Both T2D and FH+ displayed elevated postprandial TG *vs.* FH- (*P*<0.001). Acute correction of hyperglycemia during the postprandial state did not affect these group differences. Increased waist circumference and BMI were positively associated with elevated postprandial plasma palmitate appearance and oxidation.

**Conclusions/Significance:**

Postprandial plasma NEFA intolerance observed in subjects with T2D is not fully established in non-diabetic offspring of both parents with T2D, despite the presence of increased postprandial plasma TG in the later. Elevated postprandial plasma NEFA appearance and oxidation in T2D is observed despite acute correction of the exaggerated glycemic excursion in this group.

## Introduction

Offspring of two parents with type 2 diabetes (T2D) have a very high lifetime risk of developing overt T2D [1]. One of their earliest identifiable metabolic defects is increased postprandial levels of plasma triacylglycerols (**TG**) and blunted early postprandial lowering of plasma nonesterified fatty acids (NEFA) [2]. In subjects at increased risk of developing T2D, impaired postprandial reduction of NEFA by insulin may contribute to the development of insulin resistance and impaired β-cell function through increased exposure of non-adipose tissues to fatty acids [3].

We have recently shown that in offspring of both parents with T2D (FH+), plasma NEFA appearance and oxidation rate both increase significantly during intravenous infusion of heparin + Intralipid [4]. We showed that this « NEFA intolerance » was linked to defective non-oxidative plasma NEFA metabolism, i.e. possibly reduced adipose tissue NEFA storage, and was unlikely to involve a defect in insulin-mediated suppression of intracellular lipolysis. We also found that lower plasma adiponectin is associated with increased NEFA appearance during intravenous lipid infusion in humans [5], which supports the concept of a connection between adipose tissue dysfunction and increased NEFA flux to non-adipose tissues during intravenous fat administration. More recently, we demonstrated that NEFA intolerance to intravenous lipid infusion is closely associated with a lipid-induced decrease in insulin sensitivity and β-cell function *in vivo* in overweight and obese individuals [6], thus linking NEFA intolerance to enhanced susceptibility to lipotoxicity in humans.

The aim of the present study was to determine whether NEFA intolerance is present during the postprandial state, e.g. during a more physiological oral fat loading, in offspring of two parents with T2D (FH+) compared to healthy subjects without a family history of T2D (FH-), and to subjects with established but well-controlled T2D. Our hypothesis was that increased postprandial NEFA appearance and oxidation may be an early feature in non-diabetic subjects at high risk of developing T2D.

## Materials and Methods

### Ethics statement

Informed written consent was obtained from all participants in accordance with the Declaration of Helsinki and the protocol received approval from the Human Ethics Committee of the *Centre hospitalier universitaire de Sherbrooke*.

### Subjects

Thirty-six non smoking subjects aged between 18 and 60 y old, participated in these metabolic studies (see **Table 1**). Eleven healthy **FH+** subjects (defined as two biological parents with onset of diabetes after age 30 that did not necessitate insulin therapy at the time of diagnosis), thirteen healthy **FH−** subjects and twelve subjects with established T2D participated in the studies. Based on a 75 g oral glucose tolerance test [7], none of the FH+ and FH- subjects had diabetes nor were they taking any chronic medication other than stable thyroid hormone replacement therapy. They had no current medical condition known to affect lipid levels or insulin sensitivity, and had no known cardiovascular disease. T2D subjects were well-controlled (HbA_1c_<7.5%) on diet alone and/or with only one oral hypoglycaemic agent (sulfonylurea, metformin, repaglinide, nateglinide or acarbose). The women who participated were pre-menopausal and the studies were conducted during the follicular phase of their menstrual cycle.

**Table 1:** Characteristics of the participants.

	Groups			*P* *	
	FH− (n = 13)	FH+ (n = 11)	T2D (n = 12)	Group	Group difference
Age (yr)	28.2±3.1	37.1±2.9	52.1±2.6	<0.001	T2D≠others
Gender (M∶F)	7∶6	2∶9	5∶7	0.22	-
BMI (kg/m^2^)	24.8±1.3	27.3±1.8	34.2±2.0	0.001	T2D≠others
LBM (kg)	55.7±4.2	46.7±3.9	54.8±4.1	0.26	-
Waist (cm)	81.5±3.5	88.9±3.5	105.7±4.2	<0.001	T2D≠others

*P values are from one-way ANOVAs with *Scheffe*'s post-hoc test for difference between groups or from Fisher's exact test. BMI: body mass index; FH-: no family history of type 2 diabetes; FH+: offspring of both parents with type 2 diabetes; LBM: lean body mass; T2D: subjects with type 2 diabetes.

### Experimental Protocols

**Figure 1:**
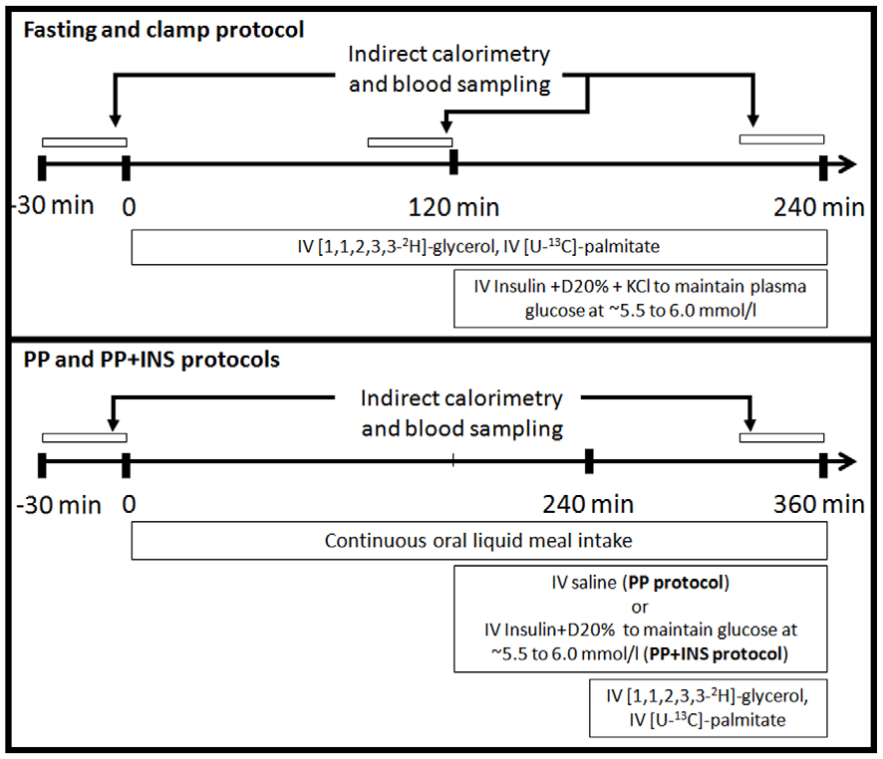
Experimental protocols. Each participant underwent three experimental protocols. The first protocol consisted, first, of a two-hour fasting experimental phase (time 0 to 120 min) followed by a two-hour normoglycemic hyperinsulinemic clamp (time 120 to 240 min) with determination of plasma glycerol and NEFA metabolism using stable isotopic tracers. The second protocol (postprandial or PP protocol) consisted in a 6-hour continuous oral intake of a standard liquid meal with determination of steady-state postprandial glycerol and NEFA metabolism using stable isotopic tracers. The third protocol (postprandial with exogenous insulin infusion or PP+INS protocol) was identical to the second protocol with the exception of intravenous insulin and dextrose infusion in the last 3 hours of the postprandial experiment to maintain normoglycemia (∼5.5 to 6.0 mmol/l) in all participants.

All subjects participated in three metabolic protocols performed 3 to 4 wk apart in random order (**Figure 1**). The subjects were instructed to follow an isocaloric diet (0% alcohol, 15% protein, 30% fat and 55% carbohydrates) for 48 h before each metabolic protocol. Subjects treated with an oral glucose lowering agent were instructed to stop their medication 2 days before the protocols and to monitor closely their blood glucose. On arrival, body weight, height and waist circumference were measured and lean body mass was determined by electrical bioimpedance (Hydra ECF/ICF, Xitron Technologies, San Diego, CA). An intravenous catheter was placed in one forearm for infusions, and another was placed in a retrograde fashion in the contralateral arm maintained in a heating box (∼55°C) for blood sampling.

The first protocol was designed to measure NEFA and glycerol metabolism at fasting for the first 2 h and during a normoglycemic (∼5.5 to 6.0 mmol/l) hyperinsulinemic clamp for the last two hours, as previously described [8] (referred to as fasting and clamp conditions throughout). The second and third protocols were designed to assess NEFA and glycerol metabolism during continuous oral intake of a standard liquid meal designed to achieve steady state postprandial NEFA and TG levels over 6 h [9] (referred to as PP condition throughout). The fat drink was prepared by sonication of soybean oil (54 g/l), safflower oil (54 g/l), dried non-fat milk (263 g/l), egg phospholipids (0.18 g/l) and water with the addition of chocolate syrup (202 g/l) and sugar (15 g/l) to provide 2465 kcal/l, 39% as fat, 16% as proteins, 45% as carbohydrates. Its fatty acid composition was similar to the composition of Intralipid, an intravenous fat emulsion that we have used in our previous studies: palmitate 10%, oleate 32%, linoleate 46%, α-linolenate 8%, and stearate 4% [9]. The oral intake of the drink corresponded to 28.4 ml every 15 min for a total of 1680 kcal over 6 h. During the third protocol, plasma glucose was clamped at ∼5.5 to 6.0 mmol/l over the last 3 h of the 6 h postprandial period using a variable dextrose 20% intravenous infusion adjusted every 5 min according to plasma glucose (Beckman Glucose Analyzer II, Beckman Instruments Corporation, Fullerton, CA) during intravenous insulin infusion (primed 0.8 mU/kg continuous 1.2 mU/kg/min infusion of Novolin Toronto, Mississauga, ON). This protocol was performed to control for any potential effect of the difference in plasma glucose on postprandial fatty acid metabolism and oxidation [10] (referred to as PP/clamp condition throughout).

From time 0 min in the fasting protocol and from time 240 min in both postprandial protocols, a constant infusion of [U-^13^C]-K palmitate (0.01 µmol/kg/min in 100 ml 25% human serum albumin; Cambridge Isotopes Laboratories Inc., Andover, MA) and a primed (1.6 µmol/kg) continuous (0.11 µmol/kg/min) infusion of [1,1,2,3,3-^2^H_5_]-glycerol (Cambridge Isotopes Laboratories Inc.) were administered [8], preceded by an intravenous bolus of [1-^13^C]-NaHCO_3_ (1.2 µmol/kg, Cambridge Isotopes Laboratories Inc.) to prime the bicarbonate pool [11]. The choice of a palmitate tracer in our experimental protocols was based on the following: 1) palmitate, oleate, and linoleate are the most prevalent NEFA in human plasma and have similar clearance rates in humans [12]; 2) a palmitate tracer was previously used to measure total NEFA turnover in humans after oral fat intake [13], [14]; and 3) NEFA appearance determined with palmitate tracer alone predicts very well NEFA appearance determined using combined palmitate and linoleate tracers during intravenous infusion of heparin + Intralipid in humans (r = 0.90, *P*<0.001) [15]. All tracers were pre-tested for sterility and non-pyrogenicity.

The protocols started with 30 min bed rest, after which blood samples were taken at 10-min intervals at baseline and during the last 30 min of each of the four experimental phases: 1) end of the first two hours of the first protocol = fasting (Fasting); 2) end of the last two hours of the first protocol = normoglycemic hyperinsulinemic clamp (Clamp); 3) end of the 6-hour postprandial protocol = PP; and 4) end of the 6-hour postprandial protocol with insulin infusion = PP/clamp. Blood was collected in tubes containing Na_2_EDTA and Orlistat (30 µg/ml, Roche, Mississauga, Canada) to prevent *in vitro* triacylglycerol lipolysis. After a 10 min equilibration, VO_2_ and VCO_2_ were measured by indirect calorimetry (Vmax29n, Sensormedics) during a 30 min baseline period and during the last 30 min of each four experimental phases to determine net carbohydrate (net CHOox) and net fatty acid oxidation (net FATox) [16]. Expiratory gases were collected at baseline and at 10-min intervals into 10 ml evacuated glass tubes (Exetainers, Labco Ltd, High Wycombe, Buckinghamshire, UK) [8].

### Laboratory assays

Glucose, insulin, total NEFA and TG were measured as described [4]. Plasma glycerol and plasma [1,1,2,3,3-^2^H_5_]-glycerol enrichment were measured by GC/MS whereas plasma palmitate, linoleate, oleate, and [U-^13^C]-palmitate enrichment were measured by LC/MS as previously described [8]. The intra-assay and inter-assay coefficients of variation were less than 15% for all of these assays. Breath ^13^CO_2_/^12^CO_2_ ratio was determined using a gas isotope ratio mass spectrometer [8] (Sercon Ltd, Crewe, Cheshire, UK).

### Calculations

Insulin sensitivity index (S_I_) was calculated from the normoglycemic hyperinsulinemic clamp data [17]. Plasma palmitate appearance and clearance rates and NEFA appearance were calculated as previously described [8], [15]. Plasma glycerol appearance rate was determined from plasma glycerol M+5 enrichment over baseline and from tracer infusion rate [18]. Fractional plasma palmitate oxidation was determined [19] with correction for the fractional acetate recovery [8]. Individual assessment of the fractional acetate recovery with an acetate tracer was not possible in the present study due to its complex design, but we found from our previous studies [4], [8] that this factor can be estimated using a linear function of time of tracer infusion (y = 0.0563+0.001359t, R^2^ = 0.99, where t is duration of tracer infusion in minutes). This relationship was not significantly affected by intravenous insulin infusion (not shown). Plasma palmitate oxidation rate was then calculated by multiplying Fox_palmitate_ by Ra_palmitate_ [8]. Palmitate non-oxidative metabolism was calculated by subtracting palmitate oxidation from palmitate appearance rate.

### Statistical Analyses

Data are expressed as mean ± SEM unless stated otherwise. Gender distribution between the groups was compared by Fisher's exact test whereas other group characteristic were compared by ANOVA with *Scheffe*'s post-hoc test. Data at baseline and during the four experimental phases (Fasting, Clamp, PP and PP/clamp) were averaged and metabolic rates were expressed in absolute terms (µmol/min). Two-way ANOVAs for repeated measures were performed with experimental phases, groups and interaction as the dependent variables and with *Scheffe*'s post-hoc test to determine the significance of group differences. These analyses were performed without and with adjustment for age, BMI, gender, waist circumference or S_I_ by ANCOVA since subjects with T2D in our study were older and more obese than the other two groups and because gender distribution was not strictly matched between groups. We also examined the relationship between postprandial glycerol and NEFA metabolic rates and age, gender, BMI, lean body mass, waist circumference, REE, glycemia and insulin sensitivity index using univariate Pearson correlations. When multiple significant correlations were found, multivariate models were built to examine whether these variables remained independently associated with postprandial glycerol and NEFA metabolic rates. A two-tailed *P* value<0.05 was considered significant. All analyses were performed with the SAS software for Windows, version 9.1.3 (SAS Institute Inc, Cary, NC).

## Results

### Basal fasting plasma metabolites and insulin (Table 2 -see also expanded version of the table in the supporting material ‘Table S1’)

**Table 2:** Basal fasting metabolites and insulin levels.

		Protocols			*P* *		
	Groups	Fasting and clamp protocol	PP	PP/clamp	Protocol	Group	Group difference
Glucose (mmol/l)	FH−	5.0±0.2	4.8±0.1	4.7±0.1	0.37	<0.001	T2D≠others
	FH+	5.0±0.1	5.0±0.1	4.8±0.1			
	T2D	7.0±0.4	6.5±0.4	7.0±0.3			
Insulin (pmol/l)	FH−	66±8	58±5	55±5	0.57	<0.001	T2D≠others
	FH+	81±8	75±6	90±10			
	T2D	123±23	127±27	166±44			
NEFA (µmol/l)	FH−	397±44	433±40	454±43	0.88	0.09	-
	FH+	471±85	392±53	374±55			
	T2D	514±46	543±72	476±75			
TG (mmol/l)	FH−	0.95±0.12	0.84±0.12	0.80±0.10	0.38	<0.001	T2D≠others
	FH+	1.25±0.22	1.12±0.15	1.12±0.17			
	T2D	1.69±0.15	1.54±0.28	1.42±0.16			
Glycerol (µmol/l)	FH−	72±5	72±5	75±5	0.95	0.001	T2D≠others
	FH+	75±11	73±10	71±7			
	T2D	94±7	98±10	91±11			

*P values are from two-way ANOVAs with *Scheffe*'s post-hoc test for difference between groups. Differences between groups remained significant after adjustment for lean body weight, gender or age. However, adjustment for BMI or waist circumference abolished group differences in insulin, triacylglycerol and glycerol levels, but not the other group differences.

FH−: no family history of type 2 diabetes; FH+: offspring of both parents with type 2 diabetes; LBM: lean body mass; NEFA: nonesterified fatty acids; PP: postprandial experimental protocol; PP/clamp: postprandial with insulin infusion experimental protocol; T2D: subjects with type 2 diabetes; TG: triacylglycerol.

Fasting plasma glucose, insulin, TG and glycerol were significantly higher in T2D vs. the other two groups (*P*<0.001), whereas fasting plasma NEFA were not significantly different between the three groups of participants. Fasting plasma glucose, insulin, NEFA, TG, glycerol, and free plasma palmitate, oleate and linoleate were also similar between all three experimental protocols.

### Plasma glucose, insulin, NEFA and TG (Figures 2, 3 and 4)

**Figure 2:**
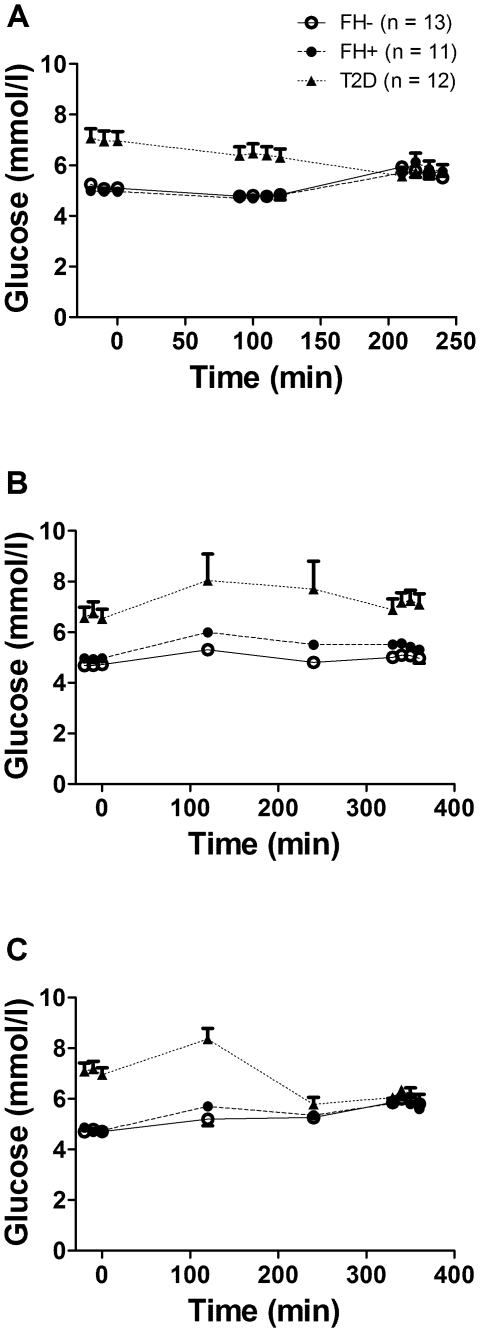
Plasma glucose over time during the experimental protocols. Plasma glucose levels during the fasting and clamp protocol (A), the postprandial protocol (B) and the postprandial protocol with exogenous insulin infusion (C) in healthy subjects without first-degree family history of type 2 diabetes (open circles, continuous lines), in offspring of both parents with type 2 diabetes (closed circles, dashed lines) and in subjects with established type 2 diabetes (closed triangles, dotted lines). Data are mean ± SEM.

**Figure 3:**
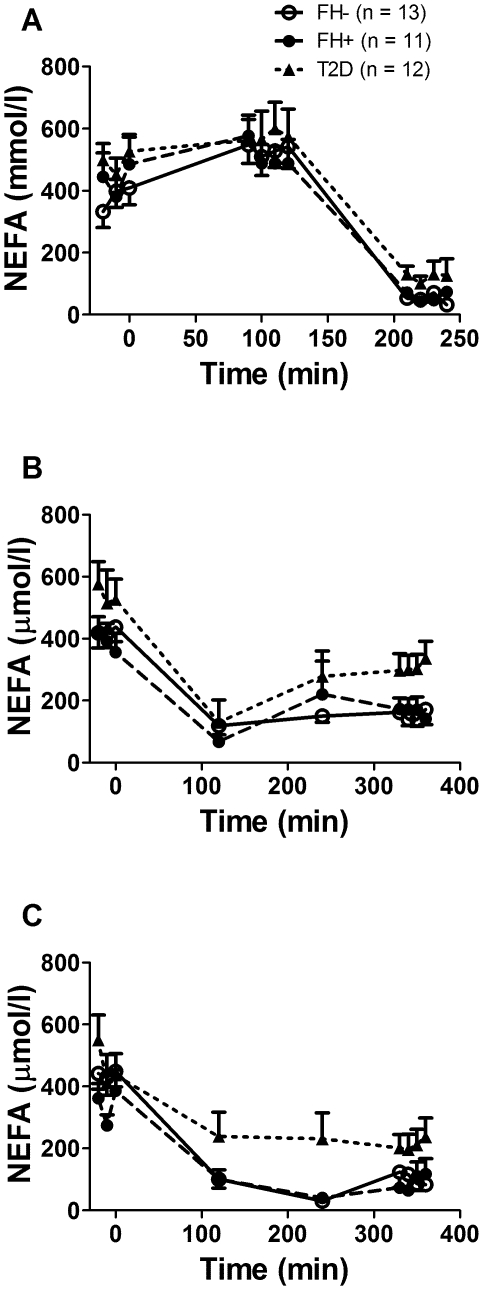
Plasma nonesterified fatty acids (NEFA) over time during the experimental protocols. Plasma nonesterified fatty acid levels during the fasting and clamp protocol (A), the postprandial protocol (B) and the postprandial protocol with exogenous insulin infusion (C) in healthy subjects without first-degree family history of type 2 diabetes (open circles, continuous lines), in offspring of both parents with type 2 diabetes (closed circles, dashed lines) and in subjects with established type 2 diabetes (closed triangles, dotted lines). Data are mean ± SEM.

**Figure 4:**
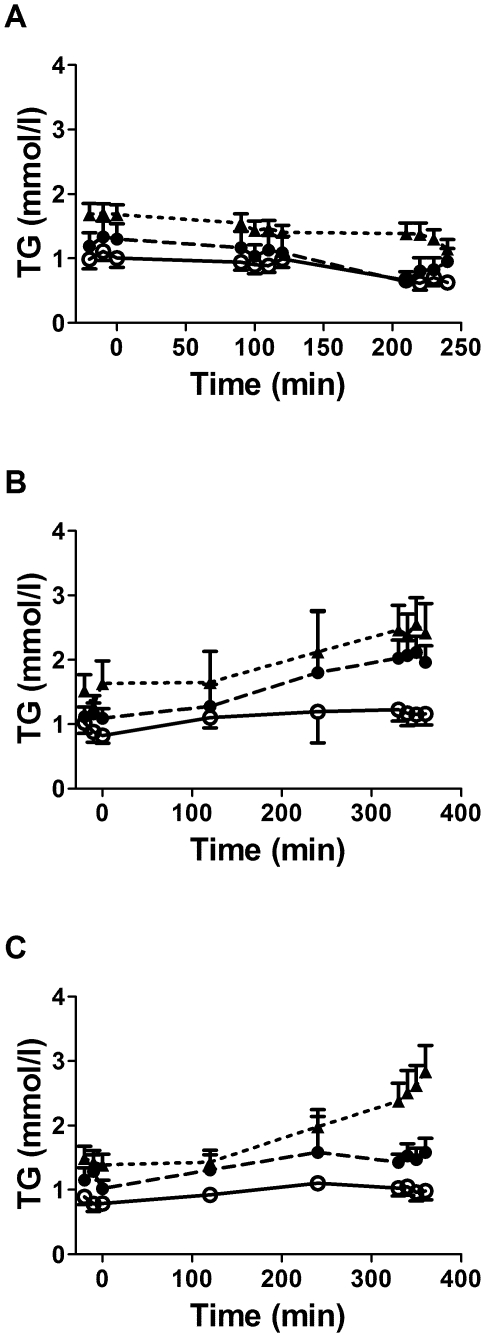
Plasma triacylglycerols (TG) over time during the experimental protocols. Plasma triacylglycerol levels during the fasting and clamp protocol (A), the postprandial protocol (B) and the postprandial protocol with exogenous insulin infusion (C) in healthy subjects without first-degree family history of type 2 diabetes (open circles, continuous lines), in offspring of both parents with type 2 diabetes (closed circles, dashed lines) and in subjects with established type 2 diabetes (closed triangles, dotted lines). Data are mean ± SEM.

Plasma glucose at fasting was significantly higher in T2D vs. FH- and FH+ (*P*<0.001) (**Figure 2A**), but was similar in all three groups during the normoglycemic hyperinsulinemic clamp. Plasma glucose throughout protocol PP (**Figure 2B**) was significantly higher in T2D (*P*<0.001), but was similar in all three groups after time 180 min in protocol PP/clamp (**Figure 2C**). Plasma insulin was higher at fasting and during PP in T2D (*P*<0.001) but was similar in the three groups during the clamp and PP/clamp (data not shown). Plasma NEFA were not significantly different between the three groups at fasting and during the clamp (**Figure 3A**), but were significantly higher in T2D (*P*<0.001) during the PP and PP/clamp protocols (**Figure 3B and 3C**). Plasma TG were higher in T2D vs. the two other groups (*P*<0.001) at fasting and during the clamp (**Figure 4A**) and in the PP and PP/clamp protocols (**Figure 4B and 4C**). Plasma TG were higher in FH+ vs. FH- (*P*<0.001) only during the PP and PP/clamp protocols. Group differences in insulin and NEFA were abolished after adjusting for waist circumference or BMI but not after adjusting for gender, age or lean body mass. Adjustment for these factors did not abolish group differences in plasma TG during the postprandial state.

### Metabolic rates during fasting and the normoglycemic hyperinsulinemic clamp (Table 3 and Figure 5 –see also expanded version of the table in the supporting material ‘Table S2’)

**Figure 5:**
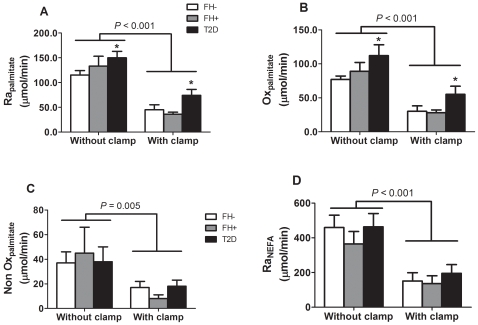
Plasma nonesterified fatty acid (NEFA) metabolism during fasting without and with euglycemic hyperinsulinemic clamp. Plasma palmitate appearance (Ra_palmitate_ – A), palmitate oxidative metabolism (Ox_palmitate_ – B), palmitate non oxidative metabolism (NonOx_palmitate_ – C) and nonesterified fatty acid appearance (Ra_NEFA_ – D) were not significantly different between healthy subjects without first-degree family history of type 2 diabetes (FH− – white bars) and offspring of both parents with type 2 diabetes (FH+ – grey bars). Subjects with established type 2 diabetes (T2D – black bars) had significantly higher Ra_palmitate_ and Ox_palmitate_ than FH−. Ra_palmitate_, Ox_palmitate_, NonOx_palmitate_ and Ra_NEFA_ were all significantly reduced during insulin clamp. * *P*<0.05 *vs.* FH−. Adjustment for age, waist circumference, BMI or insulin sensitivity, but not for gender, abolished difference between groups. Data are mean ± SEM.

**Table 3:** Metabolic rates at fasting without and with normoglycemic hyperinsulinemic clamp.

		Experimental phases		*P* *			
	Groups	No clamp	Clamp	Protocol	Group	Protocol x group	Group difference
CHOox (µmol/min)	FH−	941±128	1548±136	<0.001	0.002	0.30	T2D≠FH−
	FH+	745±130	1355±155				
	T2D	625±159	855±124				
FATox (µmol/min)	FH−	254±25	121±15	<0.001	<0.001	0.52	T2D≠others
	FH+	255±31	138±35				
	T2D	389±40	319±26				
Glycerol (µmol/l)	FH−	75±4	36±3	<0.001	0.003	0.78	T2D≠others
	FH+	77±7	35±6				
	T2D	100±10	52±8				
Ra_glycerol_ (µmol/min)	FH−	284±33	141±17	<0.001	0.01	0.83	T2D≠FH+
	FH+	235±29	141±33				
	T2D	368±53	244±61				
Palmitate (µmol/l)	FH−	145±13	21±6	<0.001	0.007	0.18	T2D≠FH−
	FH+	201±22	22±3				
	T2D	210±24	52±10				
Oleate (µmol/l)	FH−	206±15	19±2	<0.001	0.008	0.42	T2D≠FH−
	FH+	250±24	38±6				
	T2D	280±29	50±7				
Linoleate (µmol/l)	FH−	76±6	17±2	<0.001	0.005	0.13	T2D≠FH−
	FH+	107±9	20±2				
	T2D	107±12	29±3				

*P values are from two-way ANOVAs with *Scheffe*'s post-hoc test for difference between groups. Differences between groups remained significant after adjustment for gender. Adjustment for age or BMI abolished group difference in triacylglycerol and glycerol levels. Adjustment for waist circumference abolished group differences in triacylglycerol, glycerol, glycerol appearance rate and oleate levels.

CHOox: net carbohydrate oxidation rate; FATox: net fatty acid oxidation rate; FH−: no family history of type 2 diabetes; FH+: offspring of both parents with type 2 diabetes; NEFA: nonesterified fatty acids; Ra_glycerol_: glycerol appearance rate; T2D: subjects with type 2 diabetes; TG: triacylglycerol.

Net CHOox was lower whereas net FATox, plasma glycerol and individual plasma NEFA were all higher in T2D vs. FH- and FH+ (all *P*<0.005) during fasting and the clamp. Adjustment for age, BMI or waist circumference, but not for gender, abolished group differences for plasma glycerol. Glucose infusion rate and S_I_ tended to be lower in T2D vs. FH- and FH+ subjects (glucose infusion rate: 31.0±5.0, 48.3±5.5 and 45.5±6.7 µmol/kg lean body mass/min, respectively, *P* = 0.08; S_I_: 6.9±1.8, 11.1±1.2 and 9.7±1.7×10^−4^ dl/kg lean body mass/min per mU/l, respectively, *P* = 0.17). Glycerol appearance was higher in T2D *vs.* FH+ and palmitate appearance and oxidation rates were higher in T2D *vs.* FH-. Exogenous insulin infusion significantly increased net CHOox and reduced net FATox, plasma glycerol and appearance rate, individual plasma NEFA and palmitate appearance, oxidation and non oxidative disposal (all *P*<0.01). These effects of insulin infusion were similar in the three groups (all interaction *P*>0.05).

### Metabolic rates during the postprandial state without and with normalization of plasma glucose with exogenous insulin infusion (Table 4 and figure 6 –see also expanded version of the table in the supporting material ‘Table S3’)

**Figure 6:**
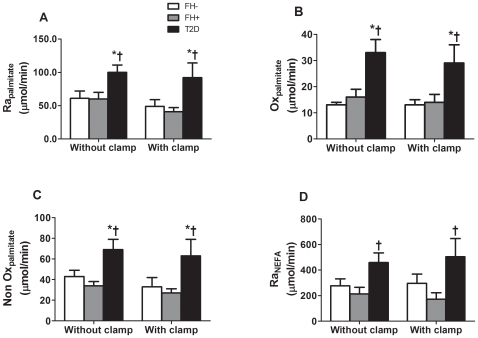
Plasma nonesterified fatty acid (NEFA) metabolism during the postprandial state without and with euglycemic hyperinsulinemic clamp. Plasma palmitate appearance (Ra_palmitate_ – A), palmitate oxidative metabolism (Ox_palmitate_ – B), palmitate non oxidative metabolism (NonOx_palmitate_ – C) and nonesterified fatty acid appearance (Ra_NEFA_ – D) in healthy subjects without first-degree family history of type 2 diabetes (FH− – white bars), offspring of both parents with type 2 diabetes (FH+ – grey bars) and subjects with established type 2 diabetes (T2D –black bars). * *P*<0.05 *vs.* FH−. † *P*<0.05 *vs.* FH+. Adjustment for waist circumference, but not for age, gender, BMI or insulin sensitivity, abolished difference between groups. Data are mean ± SEM.

**Table 4:** Metabolic rates during the postprandial state without and with normalization of glucose level with exogenous insulin infusion.

		Experimental phases		*P* *			
	Groups	PP	PP/clamp	Protocol	Group	Protocol x group	Group difference
CHOox (µmol/min)	FH−	1788±252	1863±218	0.26	0.77	0.86	-
	FH+	1680±237	1977±143				
	T2D	1577±191	1801±175				
FATox (µmol/min)	FH−	177±34	175±29	0.41	0.01	0.67	FH+≠T2D
	FH+	161±47	84±38				
	T2D	266±63	251±49				
Glycerol (µmol/l)	FH−	61±5	51±3	0.09	0.01	0.98	FH+≠T2D
	FH+	58±9	45±6				
	T2D	79±11	69±11				
Ra_glycerol_ (µmol/min)	FH−	230±26	211±31	0.54	0.006	0.95	T2D≠others
	FH+	214±46	177±41				
	T2D	339±60	329±51				
Palmitate (µmol/l)	FH−	40±10	22±6	<0.001	0.004	0.63	T2D≠others
	FH+	49±7	24±6				
	T2D	78±13	42±9				
Oleate (µmol/l)	FH−	43±4	24±2	<0.001	<0.001	0.08	FH−≠T2D
	FH+	71±10	33±4				
	T2D	96±13	43±7				
Linoleate (µmol/l)	FH−	65±10	35±5	<0.001	0.05	0.90	-
	FH+	74±8	39±5				
	T2D	95±18	55±10				

*P values are from two-way ANOVAs with *Scheffe*'s post-hoc test for difference between groups. Adjustment for gender or insulin sensitivity index did not abolish any of the group differences. Adjustment for age and BMI abolished group differences in glycerol level. Adjustment for waist circumference abolished group differences in NEFA, palmitate, oleate, linoleate, glycerol levels and appearance rate, and in net fatty acid oxidation.

CHOox: net carbohydrate oxidation rate; FATox: net fatty acid oxidation rate; FH−: no family history of type 2 diabetes; FH+: offspring of both parents with type 2 diabetes; NEFA: nonesterified fatty acids; PP: postprandial experimental protocol; PP/clamp: postprandial with insulin infusion experimental protocol; Ra_glycerol_: glycerol appearance rate; T2D: subjects with type 2 diabetes; TG: triacylglycerol.

Net CHOox tended to be lower and net FATox tended to be higher in T2D vs. the other groups during the postprandial state. Glycerol level and appearance rate, palmitate, oleate and linoleate free plasma levels and palmitate appearance (**Figure 6A**), oxidation (**Figure 6B**), non oxidative disposal (**Figure 6C**) and NEFA appearance (**Figure 6D**) were significantly higher in T2D (all *P*<0.05). Adjustment for waist circumference, but not for age, gender, BMI or S_I_, abolished these group differences. Exogenous insulin infusion significantly reduced total NEFA, palmitate, oleate and linoleate levels but did not change net CHOox and FATox, plasma glycerol and appearance rate, or palmitate appearance and oxidation and non oxidative disposal. These effects of insulin infusion were similar in the three groups (all interaction *P*>0.05).

### Determinants of plasma glycerol and NEFA metabolism during the postprandial state (Table 5 and Figure 7)

**Figure 7:**
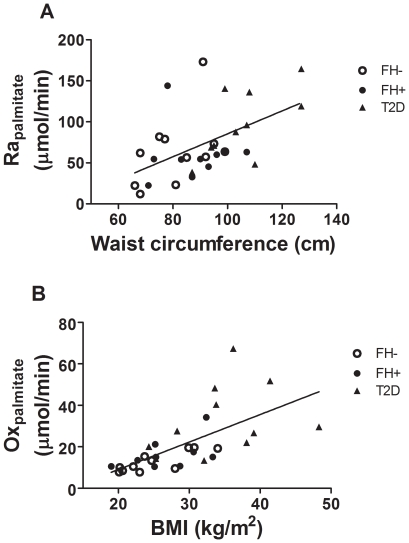
Major correlates of postprandial nonesterified fatty acid metabolism. Correlation between postprandial plasma palmitate appearance rate (Ra_palmitate_) or palmitate oxidation rate (Ox_palmitate_) and waist circumference (A) or BMI (B) in healthy subjects without first-degree family history of type 2 diabetes (FH−, open circles), in offspring of both parents with type 2 diabetes (FH+, closed circles) and in subjects with established type 2 diabetes (T2D, closed triangles).

**Table 5:** Univariate correlation between characteristics of the participants and postprandial glycerol and nonesterified fatty acid metabolism.

	Ra_glycerol_	Ra_palmitate_	Ox_palmitate_	NOx_palmitate_	FATox
Age	0.05 (0.78)	0.29 (0.09)	0.44 (0.01)	0.22 (0.24)	0.21 (0.23)
BMI	0.54 (<0.001)	0.50 (0.002)	0.77 (<0.001)	0.46 (0.01)	0.37 (0.03)
LBM	0.23 (0.18)	0.27 (0.11)	0.22 (0.24)	0.15 (0.41)	0.44 (0.008)
Waist	0.32 (0.07)	0.52 (0.002)	0.63 (<0.001)	0.47 (0.01)	0.42 (0.02)
Glycemia	−0.06 (0.74)	0.47 (0.005)	0.68 (<0.001)	0.32 (0.08)	0.30 (0.08)
S_I_	−0.36 (0.03)	−0.19 (0.27)	−0.37 (0.04)	−0.45 (0.01)	−0.25 (0.14)
REE	0.36 (0.04)	0.41 (0.02)	0.44 (0.01)	0.45 (0.01)	0.55 (<0.001)

Pearson correlation coefficients (*P* values in parenthesis) are shown between metabolic variables expressed in µmol/min. BMI: body mass index; CHOox: net carbohydrate oxidation rate; FATox: net fatty acid oxidation rate; LBM: lean body mass; NEFA: nonesterified fatty acids; nonOx_palmitate_: palmitate non oxidative metabolic rate; Ox_palmitate_: palmitate oxidation rate; Ra_glycerol_: glycerol appearance rate; Ra_NEFA_: nonesterified fatty acid appearance rate; Ra_palmitate_: palmitate appearance rate; REE: resting energy expenditure; S_I_: insulin sensitivity index.

Glycerol appearance rate was positively correlated with BMI and REE, and negatively correlated with S_I_. In multivariate analyses, BMI remained significantly and positively associated with glycerol appearance rate. Palmitate appearance rate was positively correlated with BMI, waist circumference (shown in **Figure 7A**), postprandial glycemia, and REE. Palmitate oxidation rate was positively associated with age, BMI (shown in **Figure 7B**), waist circumference, postprandial glycemia, and REE. Palmitate non oxidative metabolic rate was positively associated with BMI, waist circumference and REE. In multivariate models, waist circumference remained independently related to postprandial plasma palmitate appearance and non oxidative metabolism whereas BMI, REE and lean weight (partial R^2^ = 0.20, 0.07 and 0.07, respectively) remained independently related to palmitate oxidative rate. Net FATox was positively correlated with lean body mass, waist circumference, BMI and REE. REE remained significantly and positively associated with net FATox in multivariate analyses.

## Discussion

To our knowledge, this is the first study to report postprandial fatty acid metabolic rates using tracer techniques in FH+ subjects. We report here that FH+, a population at very high lifetime risk of developing T2D [1], do not display abnormal fasting or postprandial glycerol and NEFA metabolism compared to FH-. Plasma glycerol and NEFA metabolism were also similar between FH+ and FH- during a normoglycemic hyperinsulinemic clamp. FH+ participants in the present study had normal S_I_ and were only slightly older and overweight compared to FH- participants. In accordance with previous studies [2], however, we found that FH+ subjects display increased postprandial plasma TG compared to FH- subjects, suggesting that postprandial abnormalities in TG metabolism may occur earlier than postprandial abnormalities in NEFA metabolism in the natural history of T2D. Other investigators have shown that insulin-resistant offspring of parents with T2D have impaired skeletal muscle oxidative metabolism during fasting [20] associated with impaired muscle mitochondrial substrate oxidation [21]. The FH+ participants in the present study, in contrast, were not significantly insulin resistant compared to the FH- participants. Another previous study reported unaltered postprandial plasma NEFA levels after either a high carbohydrate or a high fat meal in FH+ subjects [22]. The latter study reported less postprandial reduction in RQ after the high fat meal in FH+ compared to FH- subjects, but did not report actual fatty acid oxidation or plasma NEFA appearance rates. We did not observe any difference in postprandial change in RQ between FH+ and FH- participants (data not shown), but our standard meal contained less fat (39% *vs.* 76% of energy content [22]).

In accordance with previous studies, we found that subjects with already established T2D displayed increased postprandial plasma NEFA and TG and increased palmitate appearance rate [13]. The T2D participants in the present study were older and more obese with large waist circumference compared to the FH- or FH+ groups. Waist circumference appeared to be a major determinant of postprandial plasma palmitate appearance in our cohort of participants, also in accordance with previous studies showing increased postprandial plasma NEFA appearance in centrally obese subjects [23]. We also demonstrated, for the first time to our knowledge, that acute correction of hyperglycemia does not alter the observed differences in postprandial plasma NEFA and glycerol metabolism between T2D and non-diabetic individuals.

Increased postprandial appearance of plasma NEFA in T2D was associated with an increase in both oxidative and non oxidative palmitate metabolism. BMI was the principal determinant of postprandial palmitate oxidation rate, whereas increased waist circumference appeared to drive the increase in non oxidative palmitate metabolism associated with T2D. We also found a significant increase in net FATox using indirect calorimetry both at fasting and in the postprandial state in participants with T2D compared to FH- and FH+ groups, results consistent with those reported from a large European cohort [24]. In the latter study, increasing BMI was associated with increasing postprandial NEFA and net FATox. Our results demonstrate using two independent methods that whole body postprandial fatty acid oxidation is elevated in T2D in humans.

In contrast to our present findings, however, many studies have shown reduced plasma palmitate oxidation rate in obese subjects and in those with impaired glucose tolerance and T2D upon stimulation of adipose tissue lipolysis with a beta-adrenergic agonist or with physical activity [19], [25], [26], associated with reduced plasma NEFA extraction and oxidation in skeletal muscle [27], [28]. In the latter *in vivo* studies, plasma NEFA and appearance rate were similar or even lower in the obese or T2D subjects. These discordant findings probably reflect both lower adipose tissue and intramyocellular lipolysis and NEFA release in insulin resistance [29]–[31], leading to reduced oxidative fatty acid utilization. We found increased plasma palmitate appearance and oxidation in T2D during the postprandial period and also during fasting, although the later differences were significant only between the T2D and FH- groups. Elevation of plasma NEFA normally leads to increased net FATox in both obese and lean men [32]. Recent studies in animal models of T2D have found results that are similar than ours with regards to circulating fatty acids (e.g. high after feeding) associated with evidence of mitochondrial fatty acid overload [33]. Some investigators have also recently challenged the presence of impaired mitochondrial oxidative function in T2D and insulin resistant subjects [34]–[38]. The results of the present study also challenge the view that impaired capacity for fatty acid oxidation occurs, at least at the whole body level, in T2D or in pre-diabetic states.

The higher oxidative disposal of NEFA in T2D observed in the present study is consistent with increased exposure to and deposition of dietary fatty acids in the liver and skeletal muscle in subjects with T2D [39]. Dietary fatty acids recycle into VLDL within hours in healthy subjects [14], [40], [41]. Indeed, some proportion of NEFA derived from intravascular TG lipolysis is available in the systemic circulation during both fasting and in the postprandial state [14], [42], [43]. Although we did not determine the source of the increased NEFA appearance and oxidation rates in T2D, we previously demonstrated that increased NEFA spillover during intravenous fat loading at high insulin level does not result from abnormal insulin-mediated suppression of intracellular lipolysis in pre-diabetic individuals [4]. It is therefore likely that enhanced postprandial NEFA appearance observed in T2D originated from dietary fatty acids.

The steady state design of our postprandial study did not fully reproduce physiological meal intake and, therefore, this may limit our conclusions. For example, in a previous study, postprandial plasma NEFA spillover was larger with discrete meal intake compared to continuous enteral infusion [40]. Therefore, it is possible that we did not detect subtle differences in postprandial NEFA metabolism in the FH+ group. Indeed, in our previous study using intravenous heparin + Intralipid infusion which induces greater and more rapid onset of fat loading in the circulation, we found significantly higher plasma NEFA and palmitate appearance rate in FH+ vs. FH- subjects of similar age, BMI and waist circumference [4]. Also supporting this interpretation is the fact that FH+ had similar postprandial NEFA levels and metabolic rates despite trend towards higher mean insulin levels (see Table S3), suggesting some adipose tissue insulin resistance in this group [44]. The design of the present study nevertheless allowed detection of significant changes in postprandial plasma NEFA metabolism in subjects with T2D. Although we cannot conclude that FH+ subjects do not display any abnormalities in postprandial NEFA metabolism, it is clear from our findings that these abnormalities are mild or not fully established compared to subjects with already established but well-controlled T2D.

We have previously demonstrated that steady state plasma NEFA levels and appearance are achieved within 90 minutes during intravenous TG emulsion without and with insulin infusion [15]. Nevertheless, postprandial circulating NEFA come from at least two different sources during the postprandial state (e.g., adipose tissue lipolysis and circulating TG lipolysis), it is therefore impossible to ascertain that both of these sources achieved steady state during the experimental design of the present study. For this reason, we opted for a 3-hour insulin infusion period during the postprandial state to maximize the likelihood of achieving steady state NEFA appearance from dietary fatty acids. Our study has other limitations. First, it was impossible to determine fractional acetate recovery in each individual and in all experimental conditions. We used a time-dependent function determined in our previous studies using intravenous heparin+Intralipid infusion without and with insulin infusion to estimate fractional acetate recovery. In support of this approach, another group recently demonstrated that application of average acetate recovery during the postprandial state leads to <10% over or underestimation of fatty acid oxidation [45]. In the latter study, it was shown that no demographic or anthropometric characteristics significantly affected acetate recovery. We therefore feel that our conclusions regarding palmitate oxidative rates are valid. Furthermore, gender distribution was not strictly matched between our groups of participants. In addition to statistical adjustment for gender that did not affect our conclusions, we also performed separate analyses including only the female participants and found similar results with regards to group differences (data not shown). Therefore, we believe that gender mismatch did not affect our conclusions. Our participants with T2D and to a lesser extent, FH+ participants, also were more obese and older than FH- participants. Although statistical adjustment for age did not generally affect group differences, adjustment for waist circumference or BMI significantly attenuated group differences in postprandial NEFA metabolism. This suggests that differences in postprandial NEFA metabolism may have been affected by varying degree of central adiposity [23], [46]. We did not measure visceral *vs.* subcutaneous abdominal fat content in our participants and, therefore, we cannot assess which central fat compartment is responsible for these associations. An increase in subcutaneous abdominal adipose tissue probably explains at least part of our findings in the participants with T2D [23], [46].

In conclusion, FH+ have increased postprandial plasma TG but do not yet fully display the increased plasma NEFA appearance and oxidation rates observed in subjects with established T2D. These abnormalities in postprandial NEFA metabolism in T2D are associated with the presence of abdominal obesity and are not prevented by acute correction of hyperglycemia. Our results suggest that increased postprandial plasma NEFA and oxidation occurs progressively over the natural history of T2D in association with the development of abdominal obesity.

## Supporting Information

Table S1 Expanded version of Table 2: Basal fasting metabolites and insulin levels. *P values are from two-way ANOVAs with Scheffe's post-hoc test for difference between groups. Differences between groups remained significant after adjustment for lean body weight, gender or age. However, adjustment for BMI or waist circumference abolished group differences in insulin, triacylglycerol and glycerol levels, but not the other group differences. CHOox: net carbohydrate oxidation rate; FATox: net fatty acid oxidation rate; FH-: no family history of type 2 diabetes; FH+: offspring of both parents with type 2 diabetes; NEFA: nonesterified fatty acids; T2D: subjects with type 2 diabetes; TG: triacylglycerol.
(0.08 MB DOC)

Table S2 Expanded version of Table 3: Metabolic rates at fasting and during normoglycemic hyperinsulinemic clamp. *P values are from two-way ANOVAs with Scheffe's post-hoc test for difference between groups. Differences between groups remained significant after adjustment for gender. Adjustment for age abolished group difference in triacylglycerol and glycerol levels and in palmitate appearance rate. Adjustment for BMI abolished group differences in triacylglycerol and glycerol levels and in glycerol and palmitate appearance rates, and in palmitate oxidation rate. Adjustment for waist circumference abolished group differences in triacylglycerol, glycerol and oleate levels, in glycerol and palmitate appearance rates and in palmitate oxidation rate. CHOox: net carbohydrate oxidation rate; FATox: net fatty acid oxidation rate; FH-: no family history of type 2 diabetes; FH+: offspring of both parents with type 2 diabetes; Fox_palmitate_: palmitate fractional oxidation rate; NEFA: nonesterified fatty acids; nonOx_palmitate_: palmitate non oxidative metabolic rate; Ox_palmitate_: palmitate oxidation rate; Ra_glycerol_: glycerol appearance rate; Ra_NEFA_: nonesterified fatty acid appearance rate; Ra_palmitate_: palmitate appearance rate; REE: resting energy expenditure; T2D: subjects with type 2 diabetes; TG: triacylglycerol, TTR: tracer to tracee ratio.
(0.13 MB DOC)

Table S3 Expanded version of Table 4: Metabolic rates during the postprandial state without and with normalization of glucose level with exogenous insulin infusion. *P values are from two-way ANOVAs with Scheffe's post-hoc test for difference between groups. Adjustment for gender or insulin sensitivity index did not abolish any of the group differences. Adjustment for age abolished group differences in glycerol level. Adjustment for BMI abolished group differences in glycerol level and appearance rate. Adjustment for waist circumference abolished group differences in NEFA, palmitate, oleate, linoleate, glycerol levels, in net fatty acid oxidation, in glycerol, palmitate and NEFA appearance rates and in palmitate oxidative and non oxidative metabolic rates. CHOox: net carbohydrate oxidation rate; FATox: net fatty acid oxidation rate; FH-: no family history of type 2 diabetes; FH+: offspring of both parents with type 2 diabetes; Fox_palmitate_: palmitate fractional oxidation rate; NEFA: nonesterified fatty acids; nonOx_palmitate_: palmitate non oxidative metabolic rate; Ox_palmitate_: palmitate oxidation rate; Ra_glycerol_: glycerol appearance rate; Ra_NEFA_: nonesterified fatty acid appearance rate; Ra_palmitate_: palmitate appearance rate; REE: resting energy expenditure; T2D: subjects with type 2 diabetes; TG: triacylglycerol, TTR: tracer to tracee ratio.
(0.13 MB DOC)
