# Supplementary material for: Increased Postprandial Nonesterified Fatty Acid Appearance and Oxidation in Type 2 Diabetes Is Not Fully Established in Offspring of Diabetic Subjects
Source: PLoS One. 2010 Jun 4;5(6):e10956. doi: 10.1371/journal.pone.0010956 (PMC2881041; doi:10.1371/journal.pone.0010956)
Supplement: Table S1 — Expanded version of Table 2: Basal fasting metabolites and insulin levels. *P values are from two-way ANOVAs with Scheffe's post-hoc test for difference between groups. Differences between groups remained significant after adjustment for lean body weight, gender or age. However, adjustment for BMI or waist circumference abolished group differences in insulin, triacylglycerol and glycerol levels, but not the other group differences. CHOox: net carbohydrate oxidation rate; FATox: net fatty acid oxidation rate; FH-: no family history of type 2 diabetes; FH+: offspring of both parents with type 2 diabetes; NEFA: nonesterified fatty acids; T2D: subjects with type 2 diabetes; TG: triacylglycerol. (0.08 MB DOC) [file pone.0010956.s001.doc]

| **Table S1. Expanded version of Table 2: Basal fasting metabolites and insulin levels** | | | | | | | |
| --- | --- | --- | --- | --- | --- | --- | --- |
|  |  | **Protocols** | | | ***P**** | | |
|  | Groups | A | B | C | Protocol | Group | Group difference |
| Glucose (mmol/l) | FH- | 5.0 ± 0.2 | 4.8 ± 0.1 | 4.7 ± 0.1 | 0.37 | < 0.001 | T2D ≠ others |
|  | FH+ | 5.0 ± 0.1 | 5.0 ± 0.1 | 4.8 ± 0.1 |  |  |  |
|  | T2D | 7.0 ± 0.4 | 6.5 ± 0.4 | 7.0 ± 0.3 |  |  |  |
| Insulin  (pmol/l) | FH- | 66 ± 8 | 58 ± 5 | 55 ± 5 | 0.57 | < 0.001 | T2D ≠ others |
|  | FH+ | 81 ± 8 | 75 ± 6 | 90 ± 10 |  |  |  |
|  | T2D | 123 ± 23 | 127 ± 27 | 166 ± 44 |  |  |  |
| NEFA  (μmol/l) | FH- | 397 ± 44 | 433 ± 40 | 454 ± 43 | 0.88 | 0.09 | - |
|  | FH+ | 471 ± 85 | 392 ± 53 | 374 ± 55 |  |  |  |
|  | T2D | 514 ± 46 | 543 ± 72 | 476 ± 75 |  |  |  |
| TG  (mmol/l) | FH- | 0.95 ± 0.12 | 0.84 ± 0.12 | 0.80 ± 0.10 | 0.38 | < 0.001 | T2D ≠ others |
|  | FH+ | 1.25 ± 0.22 | 1.12 ± 0.15 | 1.12 ± 0.17 |  |  |  |
|  | T2D | 1.69 ± 0.15 | 1.54 ± 0.28 | 1.42 ± 0.16 |  |  |  |
| Glycerol (μmol/l) | FH- | 72 ± 5 | 72 ± 5 | 75 ± 5 | 0.95 | 0.001 | T2D ≠ others |
|  | FH+ | 75 ± 11 | 73 ± 10 | 71 ± 7 |  |  |  |
|  | T2D | 94 ± 7 | 98 ± 10 | 91 ± 11 |  |  |  |
| Palmitate (μmol/l) | FH- | 122 ± 11 | 127 ± 13 | 131 ± 15 | 0.92 | < 0.001 | FH- ≠ others |
|  | FH+ | 169 ± 11 | 166 ± 19 | 169 ± 25 |  |  |  |
|  | T2D | 187 ± 21 | 178 ± 18 | 160 ± 18 |  |  |  |
| Oleate  (μmol/l) | FH- | 173 ± 12 | 176 ± 13 | 191 ± 20 | 0.87 | 0.002 | FH- ≠ T2D |
|  | FH+ | 228 ± 18 | 212 ± 19 | 208 ± 30 |  |  |  |
|  | T2D | 250 ± 24 | 251 ± 24 | 221 ± 28 |  |  |  |
| Linoleate (μmol/l) | FH- | 66 ± 5 | 67 ± 5 | 71 ± 8 | 0.94 | < 0.001 | FH- ≠ others |
|  | FH+ | 92 ± 7 | 91 ± 8 | 94 ± 11 |  |  |  |
|  | T2D | 94 ± 10 | 95 ± 9 | 81 ± 9 |  |  |  |
| CHOox (μmol/min) | FH- | 907 ± 108 | 739 ± 124 | 735 ± 122 | 0.64 | 0.23 | - |
|  | FH+ | 946 ± 178 | 951 ± 178 | 1043 ± 104 |  |  |  |
|  | T2D | 880 ± 162 | 755 ± 138 | 854 ± 154 |  |  |  |
| FATox (μmol/min) | FH- | 264 ± 21 | 340 ± 31 | 295 ± 24 | 0.24 | < 0.001 | FH+ ≠ others |
|  | FH+ | 213 ± 41 | 234 ± 64 | 171 ± 135 |  |  |  |
|  | T2D | 346 ± 33 | 364 ± 52 | 323 ± 45 |  |  |  |
