# Supplementary material for: Increased Postprandial Nonesterified Fatty Acid Appearance and Oxidation in Type 2 Diabetes Is Not Fully Established in Offspring of Diabetic Subjects
Source: PLoS One. 2010 Jun 4;5(6):e10956. doi: 10.1371/journal.pone.0010956 (PMC2881041; doi:10.1371/journal.pone.0010956)
Supplement: Table S2 — Expanded version of Table 3: Metabolic rates at fasting and during normoglycemic hyperinsulinemic clamp. *P values are from two-way ANOVAs with Scheffe's post-hoc test for difference between groups. Differences between groups remained significant after adjustment for gender. Adjustment for age abolished group difference in triacylglycerol and glycerol levels and in palmitate appearance rate. Adjustment for BMI abolished group differences in triacylglycerol and glycerol levels and in glycerol and palmitate appearance rates, and in palmitate oxidation rate. Adjustment for waist circumference abolished group differences in triacylglycerol, glycerol and oleate levels, in glycerol and palmitate appearance rates and in palmitate oxidation rate. CHOox: net carbohydrate oxidation rate; FATox: net fatty acid oxidation rate; FH-: no family history of type 2 diabetes; FH+: offspring of both parents with type 2 diabetes; Foxpalmitate: palmitate fractional oxidation rate; NEFA: nonesterified fatty acids; nonOxpalmitate: palmitate non oxidative metabolic rate; Oxpalmitate: palmitate oxidation rate; Raglycerol: glycerol appearance rate; RaNEFA: nonesterified fatty acid appearance rate; Rapalmitate: palmitate appearance rate; REE: resting energy expenditure; T2D: subjects with type 2 diabetes; TG: triacylglycerol, TTR: tracer to tracee ratio. (0.13 MB DOC) [file pone.0010956.s002.doc]

| **Table S2. Expanded version of Table 3: Metabolic rates at fasting and during normoglycemic hyperinsulinemic clamp** | | | | | | | |
| --- | --- | --- | --- | --- | --- | --- | --- |
|  |  | **Experimental phases** | | ***P**** | | | |
|  | Groups | Fasting | Clamp | Protocol | Group | Protocol x group | Group difference |
| Glucose (mmol/l) | FH- | 4.8 ± 0.1 | 5.7 ± 0.2 | 0.01 | < 0.001 | < 0.001 | T2D ≠ others |
|  | FH+ | 4.7 ± 0.1 | 5.8 ± 0.3 |  |  |  |  |
|  | T2D | 6.4 ± 0.3 | 5.7 ± 0.1 |  |  |  |  |
| Insulin  (pmol/l) | FH- | 56 ±7 | 500 ± 46 | < 0.001 | 0.16 | 0.41 | - |
|  | FH+ | 73 ± 7 | 587 ± 67 |  |  |  |  |
|  | T2D | 110 ± 18 | 791 ± 210 |  |  |  |  |
| C-peptide  (nmol/l) | FH- | 0.61 ± 0.07 | 0.93 ± 0.10 | 0.03 | 0.009 | 0.11 | T2D ≠ FH- |
|  | FH+ | 0.73 ± 0.06 | 1.16 ± 0.17 |  |  |  |  |
|  | T2D | 1.20 ± 0.11 | 1.13 ± 0.19 |  |  |  |  |
| NEFA  (μmol/l) | FH- | 540 ± 56 | 49 ± 13 | < 0.001 | 0.37 | 0.86 | - |
|  | FH+ | 506 ± 55 | 65 ± 12 |  |  |  |  |
|  | T2D | 577 ± 80 | 121 ± 31 |  |  |  |  |
| TG  (mmol/l) | FH- | 0.92 ± 0.12 | 0.66 ± 0.10 | 0.05 | < 0.001 | 0.90 | T2D ≠ others |
|  | FH+ | 1.13 ± 0.23 | 0.83 ± 0.16 |  |  |  |  |
|  | T2D | 1.44 ± 0.12 | 1.28 ± 0.15 |  |  |  |  |
| CHOox (μmol/min) | FH- | 941 ± 128 | 1548 ± 136 | < 0.001 | 0.002 | 0.30 | T2D ≠ FH- |
|  | FH+ | 745 ± 130 | 1355 ± 155 |  |  |  |  |
|  | T2D | 625 ± 159 | 855 ± 124 |  |  |  |  |
| FATox (μmol/min) | FH- | 254 ± 25 | 121 ± 15 | < 0.001 | < 0.001 | 0.52 | T2D ≠ others |
|  | FH+ | 255 ± 31 | 138 ± 35 |  |  |  |  |
|  | T2D | 389 ± 40 | 319 ± 26 |  |  |  |  |
| REE (kcal/day) | FH- | 1909 ± 98 | 1951 ± 93 | 0.11 | 0.03 | 0.05 | T2D ≠ FH+ |
|  | FH+ | 1689 ± 75 | 1776 ± 79 |  |  |  |  |
|  | T2D | 2108 ± 88 | 2077 ± 89 |  |  |  |  |
| Glycerol (μmol/l) | FH- | 75 ± 4 | 36 ± 3 | < 0.001 | 0.003 | 0.78 | T2D ≠ others |
|  | FH+ | 77 ± 7 | 35 ± 6 |  |  |  |  |
|  | T2D | 100 ± 10 | 52 ± 8 |  |  |  |  |
| TTR glycerol (%) | FH- | 4.9 ± 0.5 | 9.4 ± 1.2 | < 0.001 | 0.76 | 0.79 | - |
|  | FH+ | 4.5 ± 0.7 | 8.7 ± 1.6 |  |  |  |  |
|  | T2D | 4.8 ± 0.8 | 7.9 ± 1.4 |  |  |  |  |
| Ra_glycerol_ (μmol/min) | FH- | 284 ± 33 | 141 ± 17 | < 0.001 | 0.01 | 0.83 | T2D ≠ FH+ |
|  | FH+ | 235 ± 29 | 141 ± 33 |  |  |  |  |
|  | T2D | 368 ± 53 | 244 ± 61 |  |  |  |  |
| Palmitate (μmol/l) | FH- | 145 ± 13 | 21 ± 6 | < 0.001 | 0.007 | 0.18 | T2D ≠ FH- |
|  | FH+ | 201 ± 22 | 22 ± 3 |  |  |  |  |
|  | T2D | 210 ± 24 | 52 ± 10 |  |  |  |  |
| TTR palmitate (%) | FH- | 0.94 ± 0.06 | 3.35 ± 0.48 | < 0.001 | 0.10 | 0.12 | - |
|  | FH+ | 0.88 ± 0.13 | 3.18 ± 0.51 |  |  |  |  |
|  | T2D | 0.89 ± 0.10 | 2.11 ± 0.28 |  |  |  |  |
| Oleate (μmol/l) | FH- | 206 ± 15 | 19 ± 2 | < 0.001 | 0.008 | 0.42 | T2D ≠ FH- |
|  | FH+ | 250 ± 24 | 38 ± 6 |  |  |  |  |
|  | T2D | 280 ± 29 | 50 ± 7 |  |  |  |  |
| Linoleate (μmol/l) | FH- | 76 ± 6 | 17 ± 2 | < 0.001 | 0.005 | 0.13 | T2D ≠ FH- |
|  | FH+ | 107 ± 9 | 20 ± 2 |  |  |  |  |
|  | T2D | 107 ± 12 | 29 ± 3 |  |  |  |  |
| Ra_palmitate_ (μmol/min) | FH- | 115 ± 9 | 45 ± 10 | < 0.001 | 0.02 | 0.49 | T2D ≠ FH- |
|  | FH+ | 133 ± 20 | 36 ± 4 |  |  |  |  |
|  | T2D | 150 ± 13 | 74 ± 12 |  |  |  |  |
| FOx_palmitate_ (%) | FH- | 72 ± 6 | 69 ± 6 | 1.00 | 0.59 | 0.78 | - |
|  | FH+ | 74 ± 8 | 81 ± 6 |  |  |  |  |
|  | T2D | 75 ± 8 | 73 ± 8 |  |  |  |  |
| Ox_palmitate_ (μmol/min) | FH- | 77 ± 5 | 30 ± 8 | < 0.001 | 0.01 | 0.81 | T2D ≠ FH- |
|  | FH+ | 89 ± 13 | 28 ± 4 |  |  |  |  |
|  | T2D | 112 ± 16 | 55 ± 12 |  |  |  |  |
| nonOx_palmitate_ (μmol/min) | FH- | 37 ± 9 | 17 ± 5 | 0.005 | 0.99 | 0.68 | - |
|  | FH+ | 45 ± 21 | 8 ± 3 |  |  |  |  |
|  | T2D | 38 ± 12 | 18 ± 5 |  |  |  |  |
| Ra_NEFA_ (μmol/min) | FH- | 459 ± 71 | 151 ± 48 | < 0.001 | 0.45 | 0.81 | - |
|  | FH+ | 364 ± 72 | 136 ± 45 |  |  |  |  |
|  | T2D | 463 ± 77 | 195 ± 50 |  |  |  |  |
