# Supplementary material for: Increased Postprandial Nonesterified Fatty Acid Appearance and Oxidation in Type 2 Diabetes Is Not Fully Established in Offspring of Diabetic Subjects
Source: PLoS One. 2010 Jun 4;5(6):e10956. doi: 10.1371/journal.pone.0010956 (PMC2881041; doi:10.1371/journal.pone.0010956)
Supplement: Table S3 — Expanded version of Table 4: Metabolic rates during the postprandial state without and with normalization of glucose level with exogenous insulin infusion. *P values are from two-way ANOVAs with Scheffe's post-hoc test for difference between groups. Adjustment for gender or insulin sensitivity index did not abolish any of the group differences. Adjustment for age abolished group differences in glycerol level. Adjustment for BMI abolished group differences in glycerol level and appearance rate. Adjustment for waist circumference abolished group differences in NEFA, palmitate, oleate, linoleate, glycerol levels, in net fatty acid oxidation, in glycerol, palmitate and NEFA appearance rates and in palmitate oxidative and non oxidative metabolic rates. CHOox: net carbohydrate oxidation rate; FATox: net fatty acid oxidation rate; FH-: no family history of type 2 diabetes; FH+: offspring of both parents with type 2 diabetes; Foxpalmitate: palmitate fractional oxidation rate; NEFA: nonesterified fatty acids; nonOxpalmitate: palmitate non oxidative metabolic rate; Oxpalmitate: palmitate oxidation rate; Raglycerol: glycerol appearance rate; RaNEFA: nonesterified fatty acid appearance rate; Rapalmitate: palmitate appearance rate; REE: resting energy expenditure; T2D: subjects with type 2 diabetes; TG: triacylglycerol, TTR: tracer to tracee ratio. (0.13 MB DOC) [file pone.0010956.s003.doc]

| **Table S3. Expanded version of Table 4: Metabolic rates during the postprandial state without and with normalization of glucose level with exogenous insulin infusion** | | | | | | | |
| --- | --- | --- | --- | --- | --- | --- | --- |
|  |  | **Experimental phases** | | ***P**** | | | |
|  | Groups | PP | PP+INS | Protocol | Group | Protocol x group | Group difference |
| Glucose (mmol/l) | FH- | 5.0 ± 0.1 | 5.9 ± 0.1 | 0.55 | < 0.001 | < 0.001 | T2D ≠ others |
|  | FH+ | 5.4 ± 0.1 | 5.8 ± 0.1 |  |  |  |  |
|  | T2D | 7.1 ± 0.4 | 6.1 ± 0.2 |  |  |  |  |
| Insulin  (pmol/l) | FH- | 206 ± 32 | 951 ± 112 | < 0.001 | 0.27 | 0.91 | - |
|  | FH+ | 254 ± 33 | 1177 ± 142 |  |  |  |  |
|  | T2D | 481 ± 159 | 1354 ± 458 |  |  |  |  |
| C-peptide  (nmol/l) | FH- | 1.86 ± 0.17 | 2.72 ± 0.23 | 0.01 | 0.13 | 0.17 | - |
|  | FH+ | 2.33 ± 0.25 | 3.66 ± 0.55 |  |  |  |  |
|  | T2D | 2.73 ± 0.29 | 2.71 ± 0.51 |  |  |  |  |
| NEFA  (μmol/l) | FH- | 158 ± 28 | 102 ± 34 | 0.01 | < 0.001 | 0.77 | T2D ≠ others |
|  | FH+ | 160 ± 27 | 93 ± 29 |  |  |  |  |
|  | T2D | 306 ± 48 | 202 ± 44 |  |  |  |  |
| TG  (mmol/l) | FH- | 1.19 ± 0.17 | 1.00 ± 0.13 | 0.27 | < 0.001 | 0.56 | All different |
|  | FH+ | 2.03 ± 0.22 | 1.53 ± 0.18 |  |  |  |  |
|  | T2D | 2.45 ± 0.35 | 2.46 ± 0.30 |  |  |  |  |
| CHOox (μmol/min) | FH- | 1788 ± 252 | 1863 ± 218 | 0.26 | 0.77 | 0.86 | - |
|  | FH+ | 1680 ± 237 | 1977 ± 143 |  |  |  |  |
|  | T2D | 1577 ± 191 | 1801 ± 175 |  |  |  |  |
| FATox (μmol/min) | FH- | 177 ± 34 | 175 ± 29 | 0.41 | 0.01 | 0.67 | FH+ ≠ T2D |
|  | FH+ | 161 ± 47 | 84 ± 38 |  |  |  |  |
|  | T2D | 266 ± 63 | 251 ± 49 |  |  |  |  |
| REE (kcal/day) | FH- | 2154 ± 104 | 2430 ± 88 | < 0.001 | 0.003 | 0.10 | FH+ ≠ T2D |
|  | FH+ | 1995 ± 98 | 2059 ± 99 |  |  |  |  |
|  | T2D | 2547 ± 148 | 2677 ± 126 |  |  |  |  |
| Glycerol (μmol/l) | FH- | 61 ± 5 | 51 ± 3 | 0.09 | 0.01 | 0.98 | FH+ ≠ T2D |
|  | FH+ | 58 ± 9 | 45 ± 6 |  |  |  |  |
|  | T2D | 79 ± 11 | 69 ± 11 |  |  |  |  |
| TTR glycerol (%) | FH- | 6.6 ± 1.1 | 6.6 ± 1.0 | 0.72 | 0.44 | 0.89 |  |
|  | FH+ | 5.3 ± 0.4 | 6.1 ± 1.0 |  |  |  |  |
|  | T2D | 5.6 ± 0.8 | 5.5 ± 0.7 |  |  |  |  |
| Ra_glycerol_ (μmol/min) | FH- | 230 ± 26 | 211 ± 31 | 0.54 | 0.006 | 0.95 | T2D ≠ others |
|  | FH+ | 214 ± 46 | 177 ± 41 |  |  |  |  |
|  | T2D | 339 ± 60 | 329 ± 51 |  |  |  |  |
| Palmitate (μmol/l) | FH- | 40 ± 10 | 22 ± 6 | < 0.001 | 0.004 | 0.63 | T2D ≠ others |
|  | FH+ | 49 ± 7 | 24 ± 6 |  |  |  |  |
|  | T2D | 78 ± 13 | 42 ± 9 |  |  |  |  |
| TTR palmitate (%) | FH- | 2.26 ± 0.32 | 2.81 ± 0.45 | 0.02 | 0.08 | 0.57 | - |
|  | FH+ | 1.79 ± 0.23 | 2.94 ± 0.57 |  |  |  |  |
|  | T2D | 1.56 ± 0.17 | 2.01 ± 0.25 |  |  |  |  |
| Oleate (μmol/l) | FH- | 43 ± 4 | 24 ± 2 | < 0.001 | < 0.001 | 0.08 | FH- ≠ T2D |
|  | FH+ | 71 ± 10 | 33 ± 4 |  |  |  |  |
|  | T2D | 96 ± 13 | 43 ± 7 |  |  |  |  |
| Linoleate (μmol/l) | FH- | 65 ± 10 | 35 ± 5 | < 0.001 | 0.05 | 0.90 | - |
|  | FH+ | 74 ± 8 | 39 ± 5 |  |  |  |  |
|  | T2D | 95 ± 18 | 55 ± 10 |  |  |  |  |
| Ra_palmitate_ (μmol/min) | FH- | 61 ± 11 | 49 ± 10 | 0.25 | 0.002 | 0.94 | T2D ≠ others |
|  | FH+ | 60 ± 10 | 41 ± 6 |  |  |  |  |
|  | T2D | 100 ± 11 | 92 ± 22 |  |  |  |  |
| FOx_palmitate_ (%) | FH- | 29 ± 4 | 38 ± 5 | 0.80 | 0.16 | 0.38 | - |
|  | FH+ | 44 ± 8 | 39 ± 7 |  |  |  |  |
|  | T2D | 33 ± 4 | 32 ± 3 |  |  |  |  |
| Ox_palmitate_ (μmol/min) | FH- | 13 ± 1 | 13 ± 2 | 0.19 | < 0.001 | 0.88 | T2D ≠ others |
|  | FH+ | 16 ± 3 | 14 ± 3 |  |  |  |  |
|  | T2D | 33 ± 5 | 29 ± 7 |  |  |  |  |
| nonOx_palmitate_ (μmol/min) | FH- | 43 ± 6 | 33 ± 9 | 0.36 | 0.002 | 0.98 | T2D ≠ others |
|  | FH+ | 34 ± 4 | 27 ± 4 |  |  |  |  |
|  | T2D | 69 ± 10 | 63 ± 16 |  |  |  |  |
| Ra_NEFA_ (μmol/min) | FH- | 277 ± 54 | 296 ± 72 | 0.87 | 0.004 | 0.88 | FH+ ≠ T2D |
|  | FH+ | 213 ± 52 | 171 ± 52 |  |  |  |  |
|  | T2D | 459 ± 75 | 504 ± 143 |  |  |  |  |
